# The Designed Ankyrin Repeat Protein Antiviral Ensovibep for Nonhospitalized Patients With Coronavirus Disease 2019: Results From EMPATHY, a Randomized, Placebo-Controlled Phase 2 Study

**DOI:** 10.1093/ofid/ofae233

**Published:** 2024-05-03

**Authors:** Jeff Kingsley, Nagalingeswaran Kumarasamy, Luis Abrishamian, Marc Bonten, Awawu Igbinadolor, Martha Mekebeb-Reuter, Jennifer Rosa, Damodaran Solai Elango, Patricia Lopez, Pierre Fustier, Susana Goncalves, Charles G Knutson, Petra Kukkaro, Philippe Legenne, Krishnan Ramanathan, Shantha Rao, Evgeniya Reshetnyak, Vaia Stavropoulou, Nina Stojcheva, Michael T Stumpp, Andreas Tietz, Marianne Soergel, Richa Chandra

**Affiliations:** Centricity Research (formerly IACT Health), Columbus, Georgia, USA; VHS Infectious Diseases Medical Centre, Chennai Antiviral Research and Treatment Clinical Research Site, Chennai, India; South Bay Clinical Research Institute, Redondo Beach, California, USA; Department of Epidemiology & Health Economics, University Medical Center Utrecht, Utrecht, The Netherlands; Monroe Biomedical Research, Monroe, North Carolina, USA; Excellentis Clinical Trial Consultants, George, South Africa; Clinresco Centres, Gauteng, South Africa; Novartis Global Health, Global Drug Development, Hyderabad, India; Novartis Global Health, Novartis Pharma AG, Basel, Switzerland; Molecular Partners AG, Zurich-Schlieren, Switzerland; Novartis Global Health, Novartis Pharma AG, Basel, Switzerland; Novartis Global Health, Biomedical Research, Cambridge, Massachusetts, USA; Novartis Global Health, Novartis Pharma AG, Basel, Switzerland; Molecular Partners AG, Zurich-Schlieren, Switzerland; Novartis Global Health, Novartis Pharma AG, Basel, Switzerland; Novartis Global Health, Global Drug Development, East Hanover, New Jersey, USA; Novartis Global Health, Global Drug Development, East Hanover, New Jersey, USA; Molecular Partners AG, Zurich-Schlieren, Switzerland; Molecular Partners AG, Zurich-Schlieren, Switzerland; Molecular Partners AG, Zurich-Schlieren, Switzerland; Novartis Global Health, Novartis Pharma AG, Basel, Switzerland; Molecular Partners AG, Zurich-Schlieren, Switzerland; Novartis Global Health, Global Drug Development, East Hanover, New Jersey, USA

**Keywords:** COVID-19, DARPin, ensovibep, randomized clinical trial, SARS-CoV-2

## Abstract

**Background:**

The coronavirus disease 2019 (COVID-19) pandemic was characterized by rapid evolution of severe acute respiratory syndrome coronavirus 2 (SARS-CoV-2) variants, affecting viral transmissibility, virulence, and response to vaccines/therapeutics. EMPATHY (NCT04828161), a phase 2 study, investigated the safety/efficacy of ensovibep, a multispecific designed ankyrin repeat protein (DARPin) with multivariant in vitro activity, in ambulatory patients with mild to moderate COVID-19.

**Methods:**

Nonhospitalized, symptomatic patients (N = 407) with COVID-19 were randomized to receive single-dose intravenous ensovibep (75, 225, or 600 mg) or placebo and followed until day 91. The primary endpoint was time-weighted change from baseline in log_10_ SARS-CoV-2 viral load through day 8. Secondary endpoints included proportion of patients with COVID-19–related hospitalizations, emergency room (ER) visits, and/or all-cause mortality to day 29; time to sustained clinical recovery to day 29; and safety to day 91.

**Results:**

Ensovibep showed superiority versus placebo in reducing log_10_ SARS-CoV-2 viral load; treatment differences versus placebo in time-weighted change from baseline were −0.42 (*P* = .002), −0.33 (*P* = .014), and −0.59 (*P* < .001) for 75, 225, and 600 mg, respectively. Ensovibep-treated patients had fewer COVID-19–related hospitalizations, ER visits, and all-cause mortality (relative risk reduction: 78% [95% confidence interval, 16%–95%]) and a shorter median time to sustained clinical recovery than placebo. Treatment-emergent adverse events occurred in 44.3% versus 54.0% of patients in the ensovibep and placebo arms; grade 3 events were consistent with COVID-19 morbidity. Two deaths were reported with placebo and none with ensovibep.

**Conclusions:**

All 3 doses of ensovibep showed antiviral efficacy and clinical benefits versus placebo and an acceptable safety profile in nonhospitalized patients with COVID-19.

Continuous adaptation of the severe acute respiratory syndrome coronavirus 2 (SARS-CoV-2) represents an ongoing health challenge due to unpredictable changes in transmissibility, and virulence, evasion of natural and adaptive immunity, and variable susceptibility to antiviral therapeutics [1, 2]. Viral entry inhibition by anti-spike monoclonal antibodies showed clinical benefits, preventing disease progression and reducing all-cause mortality [3, 4]. However, the evolution of spike protein mutations in SARS-CoV-2 variants led to loss of activity of all authorized monoclonal antibodies for emergency use [5, 6].

Ensovibep is a first-in-class, multispecific DARPin (designed ankyrin repeat protein) antiviral [7, 8], composed of 5 DARPin domains: 3 bind specifically to the SARS-CoV-2 spike protein receptor-binding domain and 2 bind to human serum albumin to enable half-life extension [8]. In vitro studies demonstrated that ensovibep maintains high potency against several previous SARS-CoV-2 variants of concerns, including the Omicron subvariants BA.1 and BA.2 [8]. The Omicron subvariants BA.4 and BA.5 have a point mutation at F486V, leading to a sizable reduction in ensovibep neutralization potency [9]. Current SARS-CoV-2 variants (including JN.1) also carry mutations at position F486 of the spike protein, making them unlikely to be susceptible to ensovibep [10]. In healthy adults, ensovibep was well tolerated and showed predictable exposure confirming the expected half-life of approximately 2 weeks [11]. The National Institutes of Health–sponsored Accelerating COVID-19 Therapeutic Interventions and Vaccines-3 (ACTIV-3) trial did not demonstrate improved clinical outcomes in hospitalized patients with coronavirus disease 2019 (COVID-19) receiving standard of care and ensovibep versus placebo; no safety concerns were identified in this study [12]. In a small 2-arm phase 2a study in nonhospitalized, symptomatic patients with COVID-19, both the 225- and 600-mg doses of ensovibep were associated with rapid decline in viral load (measured by real-time polymerase chain reaction [PCR) and were well tolerated [13].

**E**nsovibep **M**ulticenter **P**lacebo-controlled study in **A**mbulatory patients with symp**T**omatic COVID-19 (p**H**ase 2 and 3 for efficac**Y** and safety) (EMPATHY; NCT04828161), a phase 2 dose-ranging study, evaluated the safety and efficacy of ensovibep in nonhospitalized patients with mild to moderate COVID-19.

## METHODS

### Trial Oversight

EMPATHY was a global, multicenter, randomized, placebo-controlled, double-blind, dose-ranging phase 2 study that evaluated the viral load reduction, clinical efficacy, safety, and tolerability of ensovibep in nonhospitalized adult patients with symptomatic COVID-19. The study was conducted and reported in accordance with the Harmonized Tripartite Guidelines for Good Clinical Practice of the International Council for Harmonisation of Technical Requirements for Pharmaceuticals for Human Use, applicable local regulations, and the ethical principles described in the Declaration of Helsinki. The study was overseen by an independent data monitoring committee that assessed, at periodic intervals, the progress of the clinical trial, emerging safety data, and critical efficacy variables.

### Patient Consent Statement

The EMPATHY study protocol was developed jointly by Molecular Partners AG and Novartis and was approved by the institutional review board and/or independent ethics committees (please refer to the Supplementary Appendix for the redacted protocol). All patients provided written informed consent before enrollment in this trial.

### Patients

Nonhospitalized adult patients who had ≥2 COVID-19 symptoms (onset within the past 7 days of dosing) and a positive SARS-CoV-2 rapid antigen test on the day of dosing, and who gave informed written consent, were included in the study. The study did not exclude patients with comorbidities (eg, renal or hepatic impairment) or on any comedications (except other SARS-CoV-2 antivirals), or after COVID-19 vaccination. In the United States (US) study sites, patients at high risk for severe COVID-19, as defined by the protocol, were excluded; high-risk patients were allowed to participate at non-US sites (please refer to the Supplementary Appendix for definition of high risk). Full inclusion and exclusion criteria as well as SARS-CoV-2 testing details can be found in the Supplementary Appendix.

### Trial Procedures

Patients were randomized (1:1:1:1) via interactive response technology to receive either ensovibep (75, 225, or 600 mg) or placebo (isotonic saline), as a single, intravenous infusion over 60 minutes (Supplementary Figure 1). Patients were stratified by their risk for developing severe COVID-19. The definition of “at high-risk” can be found in the Supplementary Appendix. All patients were followed up to day 91 postrandomization.

### Outcomes

The primary endpoint was defined as time-weighted change from baseline in log_10_ SARS-CoV-2 viral load in nasopharyngeal swab through day 8, by quantitative reverse-transcription PCR (TaqPath COVID-19 Combo Kit, ThermoFisher, with calibration curve of Armored RNA Quant SARS-CoV-2 Controls, Asuragen; see Supplementary Appendix for further details on SARS-CoV-2 antibody tests used to determine antibody positivity). Viral load reduction was chosen as the primary endpoint to assess the antiviral activity of ensovibep and dose selection for phase 3 in agreement with the US Food and Drug Administration (FDA) [14].

Secondary clinical endpoints were (1) the proportion of patients with hospitalizations and/or emergency room (ER) visits related to COVID-19, or all-cause death up to day 29; (2) time to sustained clinical recovery (defined as the first day when all symptoms from the modified 14-item FDA COVID-19 symptom list, scored as moderate or severe at baseline, were scored as mild or absent, and all symptoms from the modified FDA COVID-19 symptom list, scored as mild at baseline, were scored as absent, with no subsequent worsening, up to day 29) based on resolution of or improvement in clinical symptoms (patient-reported outcomes) up to day 29; and (3) safety analysis up to day 91, which included proportion of patients who experienced treatment-emergent adverse events (TEAEs), serious adverse events (SAEs), including death from any cause, and adverse events (AEs) of special interest (AESIs; see Supplementary Appendix for details) up to end of study (day 91). AEs were coded in accordance with the Medical Dictionary for Regulatory Activities (MedDRA).

Exploratory endpoints included patient-reported outcomes through the 36-Item Short Form Survey (SF-36) [15], and a long-COVID questionnaire (see Supplementary Appendix for details) on days 29, 61, and 91.

### Clinical Pharmacology

Modeling and simulation provided rationale for dose selection by integrating a human SARS-CoV-2 viral kinetics model with ensovibep-binding measurements and preliminary population pharmacokinetics data; a single, 75-mg intravenous infusion was projected to have a near-maximal effect [16]. Doses of 225 and 600 mg were selected to account for uncertainty in the projections and to observe an eventual dose response. Pharmacokinetics and anti-drug antibodies (ADAs) were assessed from ensovibep-treated patients and are described in the Supplementary Appendix.

### Statistical Analysis

Efficacy and safety data were presented by treatment group (ensovibep 75, 225, or 600 mg and placebo). For the primary endpoint, Multiple Comparison Procedure–Modelling (MCP-Mod) methodology (Supplementary Appendix) was employed to confirm an overall treatment-response signal, to characterize the treatment-response relationship across the 3 doses of ensovibep, and to estimate the smallest dose with clinically relevant effect over placebo. The null hypothesis of all investigational doses having the same mean as the placebo group for the time-weighted change from baseline in log_10_ SARS-CoV-2 viral load through day 8 was tested at a 1-sided 10% α level. A sample size of at least 100 patients per treatment group, for a total of 400 patients, would have at least 80% power (minimum power over the considered candidate shapes) with a 1-sided type I error rate of 0.10 to detect a dose-response trend versus placebo, using MCP-Mod to select the smallest dose for consideration.

For the secondary endpoint of proportion of patients with hospitalizations (≥ 24 hours of acute care) and/or ER visits related to COVID-19 or all-cause death up to day 29, the number and percentage of patients experiencing these events were obtained to determine relative risk versus placebo. Additionally, for each ensovibep dose versus placebo, the relative risk reduction and corresponding 95% confidence intervals (CIs) were calculated based on the relative risk parameters.

The cumulative proportions of patients who achieved sustained clinical recovery by visit days up to day 29 were estimated for each treatment group using Kaplan-Meier methods to account for losses to follow-up. A Cox proportional hazards model was employed, stratified by baseline risk of progression to severe COVID-19 and/or hospitalization (“at high risk” vs “not at high risk”).

In the safety analyses, numbers and percentages of patients with TEAEs, SAEs, safety topics of interest, AESIs, and deaths were summarized by treatment group, system organ class, preferred term, and Common Terminology Criteria for Adverse Events grade until the end of the study. In addition, TEAEs leading to discontinuation of treatment, TEAEs suspected to be study drug related, and TEAEs by ADA status were summarized. Statistical analyses were performed in SAS (version 9.6) and R (version 3.6.1) software.

## RESULTS

### Patients

**Figure 1.**
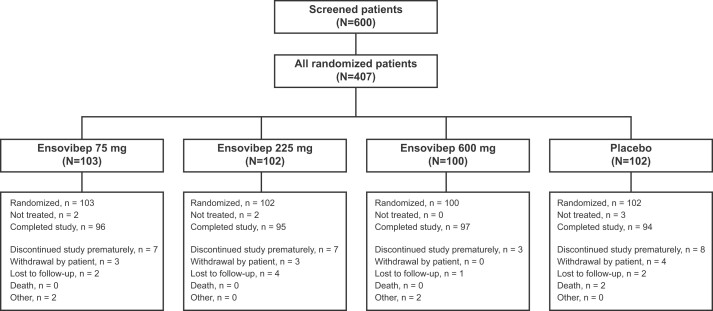
Study disposition in the **E**nsovibep **M**ulticenter **P**lacebo-controlled study in **A**mbulatory patients with sym**T**omatic COVID-19 (p**H**ase 2 and 3 for efficac**Y** and safety) (EMPATHY) study. Patients could discontinue from study treatment but continue participating in the study. Two patients randomized to ensovibep 75 mg did not receive the treatment they were randomized to: 1 due to screening failure and the other was denied for dosing after randomization. Two patients randomized to ensovibep 225 mg did not receive the treatment they were randomized to: 1 patient received no active study drug because the infusion bag was not prepared correctly, and 1 patient received a lower dose (<75 mg) as the infusion was interrupted. For the safety set, these 2 patients were analyzed in the placebo and ensovibep 75 mg arms, respectively. In the placebo arm, 3 patients did not receive the treatment they were randomized to: 1 patient was never dosed due to randomization failure, and 2 withdrew consent before dosing.

In total, 600 patients were screened and 407 were randomized 1:1:1:1 to ensovibep 75, 225, and 600 mg and placebo at 48 sites in the US, South Africa, India, The Netherlands, and Hungary from 10 May to 21 October 2021. Of these patients, a total of 400 received treatment (Figure 1).

Baseline characteristics were well balanced across treatment groups (Table 1). The median age of the study population was 41.0 years (range, 18–81 years); 45.5% were male and 62% were White. The ethnographic distribution reflected the location of study sites, with most patients being enrolled in the US. Most patients (334/400 [83.5%]) randomized were not at high risk of developing severe COVID-19 infection, and 66 of 400 (16.5%) were at “high risk” as per the protocol definition. However, due to an update of the FDA “high-risk” definition (primarily lowering the body mass index threshold from 35 to 25 kg/m^2^), in May 2021 while the study was ongoing [17], 298 of 400 patients (74.5%) met the new definition for “high risk” for COVID-19 progression. Baseline SARS-CoV-2 sequencing indicated that Delta was the predominant variant (B.1.617.2; 322/400 [80.5%]), with a similar distribution across the ensovibep and placebo arms. Seventy-four of 400 (18.5%) patients had at least 1 dose of an approved SARS-CoV-2 vaccine; SARS-CoV-2 antibodies (immunoglobulin G [IgG] and immunoglobulin M [IgM]) against spike protein were present at baseline in 194 of 386 patients (50.3%), including 62 vaccinated patients. The proportion of patients positive for anti-spike IgG, IgM, and virus neutralizing antibodies at baseline and day 91 are detailed in Supplementary Table 1.

**Table 1.** Baseline Demographics and Clinical Characteristics

Characteristic Statistic/Category	Ensovibep 75 mg	Ensovibep 225 mg	Ensovibep 600 mg	Ensovibep Total	Placebo	All
	(n = 101)	(n = 100)	(n = 100)	(n = 301)	(n = 99)	(n = 400)
Age, y, median (range)	41.0 (19–70)	39.0 (19–66)	41.0 (18–71)	40.0 (18–71)	41.0 (18–81)	41.0 (18–81)
Age group, y						
<25	8 (7.9)	11 (11.0)	7 (7.0)	26 (8.6)	10 (10.1)	36 (9.0)
25–44	52 (51.5)	51 (51.0)	56 (56.0)	159 (52.8)	49 (49.5)	208 (52.0)
45–64	37 (36.6)	37 (37.0)	35 (35.0)	109 (36.2)	35 (35.4)	144 (36.0)
**≥**65	4 (4.0)	1 (1.0)	2 (2.0)	7 (2.3)	5 (5.1)	12 (3.0)
Male sex	41 (40.6)	46 (46.0)	53 (53.0)	140 (46.5)	42 (42.4)	182 (45.5)
Geographical region						
Asia	13 (12.9)	12 (12.0)	12 (12.0)	37 (12.3)	12 (12.1)	49 (12.3)
Europe	7 (6.9)	5 (5.0)	4 (4.0)	16 (5.3)	6 (6.1)	22 (5.5)
North America	59 (58.4)	61 (61.0)	63 (63.0)	183 (60.8)	59 (59.6)	242 (60.5)
Africa	22 (21.8)	22 (22.0)	21 (21.0)	65 (21.6)	22 (22.2)	87 (21.8)
Race						
White	62 (61.4)	63 (63.0)	59 (59.0)	184 (61.1)	63 (63.6)	247 (61.8)
Black or African American	14 (13.9)	11 (11.0)	16 (16.0)	41 (13.6)	11 (11.1)	52 (13.0)
Asian	13 (12.9)	14 (14.0)	14 (14.0)	41 (13.6)	16 (16.2)	57 (14.3)
Native Hawaiian or other Pacific Islander	2 (2.0)	1 (1.0)	1 (1.0)	4 (1.3)	0	4 (1.0)
American Indian or Alaska Native	1 (1.0)	0	3 (3.0)	4 (1.3)	0	4 (1.0)
Multiple	6 (5.9)	4 (4.0)	5 (5.0)	15 (5.0)	8 (8.1)	23 (5.8)
Unknown	0	3 (3.0)	1 (1.0)	4 (1.3)	0	4 (1.0)
Not reported	3 (3.0)	4 (4.0)	1 (1.0)	8 (2.7)	1 (1.0)	9 (2.3)
Weight, kg, median (range)	75.0 (40.8–131.7)	80.0 (53.6–130.0)	76.5 (46.3–183.3)	76.40 (40.8–183.3)	75.4 (42.2–132.0)	76.4 (40.8–183.3)
BMI, kg/m**^2^**, median (range)	26.7 (16.7–52.1)	28.2 (18.8–45.6)	26.8 (17.9–59.2)	26.9 (16.7–59.2)	26.6 (17.8–46.2)	26.9 (16.7–59.2)
BMI category, kg/m^2^						
<18.5	2 (2.0)	0	1 (1.0)	3 (1.0)	1 (1.0)	4 (1.0)
18.5–24.9	32 (31.7)	26 (26.0)	33 (33.0)	91 (30.2)	34 (34.3)	125 (31.3)
25–34.9	61 (60.4)	67 (67.0)	59 (59.0)	187 (62.1)	62 (62.6)	249 (62.3)
≥35	6 (5.9)	7 (7.0)	7 (7.0)	20 (6.6)	2 (2.0)	22 (5.5)
Risk for COVID-19 disease progression						
High risk (protocol definition)	22 (21.8)	16 (16.0)	15 (15.0)	53 (17.6)	13 (13.1)	66 (16.5)
High risk (updated FDA definition**^a^**)	75 (74.3)	78 (78.0)	73 (73.0)	226 (75.1)	72 (72.7)	298 (74.5)
Log_10_ baseline SARS-CoV-2 viral load						
No.	84	90	86	260	87	347
Median (min–max)	6.7 (3.3–8.5)	6.9 (2.9–8.7)	6.7 (2.7–8.7)	6.7 (2.7–8.7)	6.4 (2.7–8.6)	6.7 (2.7–8.7)
**≥**6	63 (62.4)	62 (62.0)	61 (61.0)	186 (61.8)	52 (52.5)	238 (59.5)
SARS-CoV-2 antibodies at baseline						
Present	50 (49.5)	52 (52.0)	47 (47.0)	149 (49.5)	45 (45.5)	194 (48.5)
SARS-CoV-2 variant						
Delta/B.1.617.2	78 (77.2)	82 (82.0)	82 (82.0)	242 (80.4)	80 (80.8)	322 (80.5)
Other (WT, Alpha, Beta, Gamma)	6 (6.0)	5 (5.0)	8 (7.9)	21 (7.0)	5 (5.1)	24 (6.0)
Missing	13 (12.9)	13 (13.0)	12 (12.0)	38 (12.6)	14 (14.1)	52 (13.0)
Baseline COVID-19 disease severity						
Mild	67 (66.3)	61 (61.0)	61 (61.0)	189 (62.8)	70 (70.7)	259 (64.8)
Moderate	33 (32.7)	39 (39.0)	38 (38.0)	110 (36.5)	29 (29.3)	139 (34.8)
Severe	1 (1.0)	0	1 (1.0)	2 (0.7)	0	2 (0.5)

Data are presented as No. (%) unless otherwise indicated.

Abbreviations: BMI, body mass index; COVID-19, coronavirus disease 2019; FDA, Food and Drug Administration; SARS-CoV-2, severe acute respiratory syndrome coronavirus 2; WT, wild type.

^a^Patients with BMI ≥35 kg/m^2^ were considered high risk per protocol definition. According to the updated definition of “high risk” issued by the FDA in May 2021, BMI between 25 and 35 kg/m^2^ was also considered high risk.

### Efficacy

For the primary endpoint, a statistically significant treatment-response difference over placebo was established using MCP-Mod methodology. The null hypothesis of all investigational doses having the same mean change as the placebo group (referred to as flat dose response) was rejected (Supplementary Figure 2). The dose-signal shape with the highest test statistic was the maximum effect (median effective dose [ED_50_] = 40) shape (*P* < .001).

**Figure 2.**
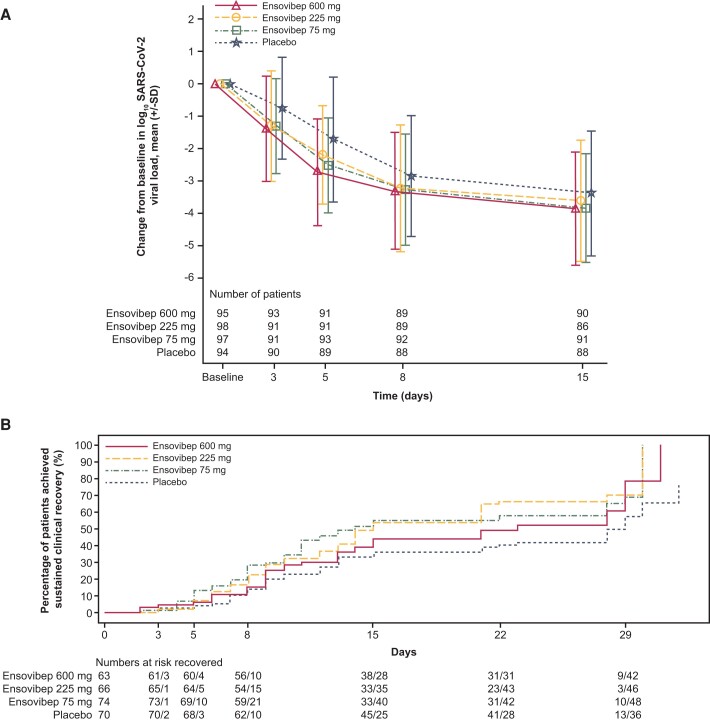
*A*, Mean change from baseline in viral load to day 15 with ensovibep versus placebo. Vertical bars represent standard deviation. *B*, Kaplan-Meier curve for the time to sustained clinical recovery in patients treated with ensovibep versus placebo up to day 29 (full analysis set). The full analysis set includes all patients in the randomized set for whom intravenous infusion of study treatment was initiated during the treatment period, excluding misrandomized patients. Abbreviations: SARS-CoV-2, severe acute respiratory syndrome coronavirus 2; SD, standard deviation.

Ensovibep showed statistically significant treatment differences versus placebo in the time-weighted change from baseline in log_10_ SARS-CoV-2 viral load through day 8 in the adjusted analysis of covariance (ANCOVA) model: −0.42 for the 75-mg arm (95% CI, −.68 to −.15; *P*= .002), −0.33 for the 225-mg arm (95% CI, −.60 to −.07; *P* = .014), and −0.59 for the ensovibep 600-mg arm (95% CI, −.86 to −.32; *P* < .001). In the subgroup of patients at high risk of progression to severe COVID-19, larger viral load reductions were observed with ensovibep versus placebo (Supplementary Table 2). Treatment differences were higher in patients who had a baseline log_10_ SARS-CoV-2 viral load ≥6 versus those with baseline log_10_ SARS-CoV-2 viral load <6 (Supplementary Table 2). Absolute viral load reduction from baseline through day 15 was greater for all 3 doses of ensovibep compared with placebo (Figure 2*A*). The robustness and consistency of the primary analysis of time-weighted change in viral load (using ANCOVA) and treatment-response signal (using MCP-Mod methodology) were confirmed by multiple predefined supplemental and supportive analyses. Ensovibep was found to be effective at reducing SARS-CoV-2 viral load regardless of the presence or absence of anti-SARS-CoV-2 antibodies prior to treatment (Supplementary Figure 3).

Fewer COVID-19–related hospitalizations and/or ER visits and all-cause mortality versus placebo were observed with ensovibep at all doses up to day 29 (Table 2). No deaths were reported in any of the 3 ensovibep arms, while 2 deaths due to COVID-19 pneumonia were reported in the placebo arm (Table 2).

**Table 2.** Patients With Hospitalization and/or Emergency Room Visits Related to Coronavirus Disease 2019 or Death From Any Cause With Ensovibep at All Doses Versus Placebo (up to Day 29)^a^

Event	Ensovibep 75 mg	Ensovibep 225 mg	Ensovibep 600 mg	Ensovibep Total	Placebo
	(n = 101)	(n = 100)	(n = 100)	(n = 301)	(n = 99)
Any event	0	3 (3.0)	1 (1.0)	4 (1.3)	6 (6.1)
Hospitalizations (**≥**24** **h of acute care)	0	2 (2.0)	0	2 (0.7)	5 (5.1)^b^
ER visits related to COVID-19	0	1 (1.0)	1 (1.0)	2 (0.7)	5 (5.1)^b^
Death from any cause	0	0	0	0	2 (2.0)^c^
Relative risk reduction (ensovibep vs placebo)	…	…	…	0.78	…
95% CI	…	…	…	.16–.95	…
*P* value	…	…	…	.017	…
Absolute risk difference (ensovibep vs placebo)	…	…	…	−4.7	…
95% CI	…	…	…	−11.6 to −.5	…
*P* value	…	…	…	.017	…

Data are presented as No. (%) unless otherwise indicated.

Abbreviations: CI, confidence interval; COVID-19, coronavirus disease 2019; ER, emergency room.

^a^In the hierarchy of ER visit, hospitalization, or death, patients are counted in the highest category; ER visits exclude those resulting in hospitalization/death; hospitalizations exclude those that resulted in death.

^b^Of the 6 patients from the placebo arm who reported any event, 5 had ER visits and 4 were subsequently hospitalized; 1 additional patient was directly hospitalized.

^c^Two deaths due to COVID-19 pneumonia were reported in the placebo arm.

Because no meaningful differences in viral load reduction were observed among the ensovibep arms, all 3 were pooled for the analysis of the secondary endpoint of COVID-19–related hospitalization, and/or ER visits, or all-cause mortality. The combined events were reported in 4 of 301 (1.3%) and 6 of 99 (6.1%) patients in the pooled ensovibep and placebo arms, respectively, while COVID-19–related hospitalizations were recorded in 2 of 301 (0.7%) and 5 of 99 patients (5.1%), respectively (Table 2). The relative risk reduction for experiencing hospitalizations and/or ER visits related to COVID-19 or death due to any cause was 78% (95% CI, 16%–95%; nominal *P* = .017) for the ensovibep arms compared with placebo. Excluding the ER visits, the relative risk reduction for hospitalization or death was 87% (95% CI, 34%–99%; nominal *P* = .012) for the ensovibep arms compared with placebo.

The median times to sustained clinical recovery were shorter for ensovibep versus placebo: 14, 15, and 23 days in the ensovibep 75-, 225-, and 600-mg arms, respectively, versus 29 days in the placebo arm (Figure 2*B*). The Kaplan-Meier analysis of time to sustained clinical recovery for each of the 3 ensovibep and placebo arms indicated a higher probability of recovery, by day 29, on ensovibep versus placebo. The log-rank *P* values for comparison of each dose versus placebo were .018, .008, and .08 for 75, 225, and 600 mg, respectively. The estimated cumulative proportions of patients attaining sustained clinical recovery by day 15 were 55.0%, 53.8%, 44.4%, and 36.2% in the ensovibep 75, 225, and 600 mg and placebo arms, respectively.

In the stratified Cox analysis, the rates of sustained clinical recovery were similar for ensovibep 75 mg and 225 mg and statistically higher than placebo, but not for 600 mg. By day 29, sustained clinical recovery was achieved in 66.2% (hazard ratio [HR], 1.65 [95% CI, 1.05–2.57]), 71.2% (HR, 1.65 [95% CI, 1.05–2.58]), and 70.0% (HR, 1.42 [95% CI, .90–2.25]) of patients in the ensovibep 75-, 225-, and 600-mg arms, compared to 55.6% of patients in the placebo arm. At days 29, 61, and 91, assessment of symptoms post–acute disease by the SF-36 [15] (completion rate: 323/400 [80.8%]) and long-COVID questionnaires (completion rate: 345/400 [86.3%]) showed no significant differences between the ensovibep and placebo arms (Supplementary Tables 3 and 4).

### Ensovibep Pharmacokinetics

The pharmacokinetic parameters showed that ensovibep concentration increased approximately proportionally with dose, and the observed mean half-life ranged from 12.6 to 13.8 days across dose groups (Supplementary Table 5; Supplementary Figure 4). The ensovibep mean serum concentration in the 75-mg arm was 15.3 µg/mL at day 8. This concentration was >1000-fold in excess of the cellular neutralization potency (half maximal effective concentration [EC_50_]) of ensovibep for the Delta variant, which was the predominant variant observed in EMPATHY.

### Safety

No unexpected safety findings were detected. The administration of study medication was stopped prematurely in 1 ensovibep patient because of a nonserious infusion-related reaction and in none of the placebo patients. TEAEs were reported in 133 of 300 (44.3%) and 54 of 100 patients (54.0%) in the pooled ensovibep and placebo arms, respectively. The incidence of overall TEAEs was similar in the ensovibep 600 mg and placebo arms and lowest in the ensovibep 75-mg arm (Table 3). The most common TEAEs (≥3% of patients) in the pooled ensovibep versus placebo arms were increased blood creatinine (4.3% vs 6.0%), increased alanine aminotransferase (3.7% vs 2.0%), increased fibrin D dimer (3.7% vs 3.0%), worsening of COVID-19 (3.3% vs 4.0%), increased aspartate aminotransferase (3.3% vs 2.0%), nasopharyngitis (3.0% vs 5.0%), and increased lipase (3.0% vs 1.0%) (Supplementary Table 6). Most of the grade 3 TEAEs reported were consistent with COVID-19 morbidity; no grade 4 or 5 events were reported among patients on ensovibep, while 2 cases of COVID-19–related fatal pneumonia occurred in the placebo arm.

**Table 3.** Overview of Adverse Events (Safety Set; up to Day 91)

MedDRA Primary System Organ Class	Ensovibep 600 mg	Ensovibep 225 mg	Ensovibep 75 mg	Ensovibep Total	Placebo
	(n = 100)	(n = 98)	(n = 102)	(n = 300)	(n = 100)
Patients with at least 1 TEAE	51 (51.0)	42 (42.9)	40 (39.2)	133 (44.3)	54 (54.0)
TEAEs at the maximum CTCAE grade severity					
Mild (grade 1)	26 (26.0)	19 (19.4)	16 (15.7)	61 (20.3)	25 (25.0)
Moderate (grade 2)	24 (24.0)	15 (15.3)	21 (20.6)	60 (20.0)	21 (21.0)
Severe (grade 3)	1 (1.0)	8 (8.2)	3 (2.9)	12 (4.0)	6 (6.0)
Life-threatening (grade 4)	0	0	0	0	0
Fatal (grade 5)	0	0	0	0	2 (2.0)
TEAEs suspected to be related to study medication	12 (12.0)	9 (9.2)	11 (10.8)	32 (10.7)	9 (9.0)
TEAEs leading to discontinuation of study treatment	0	0	1 (1.0)	1 (0.3)	0
Serious TEAEs	0	2 (2.0)	1 (1.0)	3 (1.0)	9 (9.0)
AESIs	20 (20.0)	9 (9.2)	7 (6.9)	36 (12.0)	18 (18.0)

Data are presented as No. (%). The safety set includes all patients who initiated intravenous infusion of study treatment regardless of being randomized or not.

Abbreviations: AESI, adverse event of special interest; CTCAE, Common Terminology Criteria for Adverse Events; MedDRA, Medical Dictionary for Regulatory Activities; TEAE, treatment-emergent adverse event.

AESIs were reported in 12.0% and 18.0% of the ensovibep and placebo arms, respectively (Supplementary Table 7). Hepatic events, including investigations, were the most commonly reported AESIs in both the ensovibep (3.3%) and placebo (5.0%) arms. These were mild, transient, nonserious, asymptomatic liver enzyme elevations, with higher incidence in the ensovibep 600-mg arm. The incidence of hypersensitivity-related events (other than administration-site reactions) reported as AESIs was comparable among treatment groups. Three cases of rash (1%), all of moderate severity (grade 2), were reported in the ensovibep arms (600 mg: n = 2; 75 mg: n = 1). One case of acute respiratory failure (1%) was reported as an AESI of worsening of COVID-19 (not associated with hypersensitivity) in the placebo arm.

ADA data at baseline and days 15, 29, 61, and 91 are detailed in Supplementary Table 8. ADAs were detected at the first assessment after administration (day 15) in 44.6%–50.0% of patients treated with ensovibep. The pooled ensovibep-treated population exhibited the highest incidence at day 61 (68.6%) with a slight subsequent decline at day 91 (64.3%). ADAs were less frequently detected with the 75-mg dose than with the 225-mg and 600-mg doses at all corresponding assessment times. The incidence of TEAEs was 31.6% and 35.5% in ADA-positive and ADA-negative patients, respectively.

## DISCUSSION

Results from this first randomized, controlled trial with a DARPin antiviral therapeutic demonstrate virological and clinical efficacy of the 3 tested ensovibep doses in patients with mild to moderate COVID-19. The study met its primary objective in demonstrating the superiority of ensovibep at all doses, compared with placebo, in reducing SARS-CoV-2 viral load, irrespective of the presence or absence of anti-SARS-CoV-2 antibodies at baseline. COVID-19–related hospitalizations and/or ER visits or all-cause mortality were also lower for patients treated with ensovibep versus placebo. The median time to sustained clinical recovery for all ensovibep arms was at least a week shorter than placebo.

The safety and tolerability profile of ensovibep was consistent with data from early clinical studies [11–13]. The incidence of hypersensitivity-related events, including rash, was similar among the treatment and placebo groups (1% vs 1%). The study data suggest that the presence of ADA did not impact the safety and/or efficacy profile of ensovibep. Postdose ADA assessments before day 15 were not performed; however, the significant viral load reductions through day 8 observed with all ensovibep doses compared with placebo suggested that ADA, if present, did not impact efficacy assessments. The incidence of AEs did not appear different between patients with and without ADAs.

The reported study results, generated during the COVID-19 pandemic Delta wave, demonstrate virological and clinical efficacy of ensovibep comparable to those reported for monoclonal antibodies. Shortly after completion of the study, new SARS-CoV-2 variants started to consistently exhibit mutations at F486, which have had a major impact on ensovibep’s activity against current variants.

The interpretation of the study data is limited by the small sample size typical for phase 2 trials; the fact that the study was conducted prior to the emergence of Omicron variants when the Delta variant was the predominant strain; and by the underrepresentation of some ethnic groups (about two-thirds of patients were White). Since the COVID-19 pandemic has disproportionally affected ethnic minorities and resource-limited regions, future COVID-19 studies should aim to assess outcomes in diverse patient populations. Also, the presented results should be interpreted in light of methodological confounders, the SARS-CoV-2 variant evolution, and increased immunity against SARS-CoV-2 variants due to exposure to the virus or vaccination.

We did not conduct a confirmatory phase 3 study due to ensovibep's lack of efficacy against new variants, a change in overall disease severity, and the evolving treatment landscape for COVID-19. However, the fact that the DARPin molecule, ensovibep, showed in vitro neutralization potency against most earlier variants of concern [8, 9], where several other anti-spike protein therapeutics had lost activity, supports the importance of multispecific and cooperative spike protein binding, and offers an alternative therapeutic platform to antibody-based spike protein neutralization for future public health emergencies and pandemics.

## Supplementary Material

ofae233_Supplementary_Data

## Supplementary Data

Supplementary materials are available at *Open Forum Infectious Diseases* online. Consisting of data provided by the authors to benefit the reader, the posted materials are not copyedited and are the sole responsibility of the authors, so questions or comments should be addressed to the corresponding author.

## Notes

***Acknowledgments.*** The authors thank the patients enrolled in this study and their families, as well as all of the participating investigators and their site teams. The authors also thank Khalida Rizi, Cathy McDonnell, and Rahul Lad of Novartis for providing medical writing support/editorial support, which was funded by Novartis Pharma AG, in accordance with Good Publication Practice guidelines for company-sponsored biomedical research (https://www.acpjournals.org/doi/10.7326/M22-14601460).

***Data sharing.*** Novartis is committed to sharing, with qualified external researchers, access to patient-level data and supporting clinical documents from eligible studies. These requests are reviewed and approved by an independent review panel on the basis of scientific merit. All data provided are anonymized to respect the privacy of patients who have participated in the trial in line with applicable laws and regulations. This trial data availability is according to the criteria and process described on www.clinicalstudydatarequest.com.
