# Supplementary material for: The Designed Ankyrin Repeat Protein Antiviral Ensovibep for Nonhospitalized Patients With Coronavirus Disease 2019: Results From EMPATHY, a Randomized, Placebo-Controlled Phase 2 Study
Source: Open Forum Infect Dis. 2024 May 3;11(6):ofae233. doi: 10.1093/ofid/ofae233 (PMC11160321; doi:10.1093/ofid/ofae233)
Supplement: ofae233_Supplementary_Data [file ofae233_supplementary_data.docx]

**The DARPin antiviral ensovibep for** **non-hospitalized patients with COVID-19: Results from EMPATHY, a randomized, placebo-controlled Phase 2 study**

**Authors:** Jeff Kingsley, Nagalingeswaran Kumarasamy, Luis Abrishamian, Marc Bonten, Awawu Igbinadolor, Martha Mekebeb-Reuter, Jennifer Rosa, Damodaran Solai Elango, Patricia Lopez, M.D., Pierre Fustier, Susana Goncalves, Charles G. Knutson, Petra Kukkaro, Philippe Legenne, Krishnan Ramanathan, Shantha Rao, Evgeniya Reshetnyak, Vaia Stavropoulou, Nina Stojcheva, Michael T. Stumpp, Andreas Tietz, Marianne Soergel, Richa Chandra.

**Supplementary appendix**

Contents

[List of investigators 3](#_Toc159928393)

[Methods 7](#_Toc159928394)

[Inclusion and exclusion criteria 7](#_Toc159928395)

[Per-protocol definition of high-risk patients 10](#_Toc159928396)

[SARS-CoV-2 and antibody testing 10](#_Toc159928397)

[Multiple Comparison Procedure – Modelling (MCP-Mod) methodology 12](#_Toc159928398)

[Adverse events of special interest (AESI) 12](#_Toc159928399)

[Long COVID-19 questionnaire 12](#_Toc159928400)

[Immunogenicity 15](#_Toc159928401)

[Pharmacokinetics 15](#_Toc159928402)

[Supplemental Figures 15](#_Toc159928403)

[Figure S1. EMPATHY study design 15](#_Toc159928404)

[Figure S2. Least squares means with 95% confidence intervals and fitted average model (median and 95% percentile interval) for time-weighed change from baseline in log_10_ SARS-CoV-2 viral load through Day 8 (full analysis set) 17](#_Toc159928405)

[Figure S3. Forest plot of estimated treatment differences and associated 95% confidence intervals in time-weighted change from baseline in log_10_ SARS-CoV-2 viral load through Day 8 by subgroups for presence of anti–SARS-CoV-2 antibodies 18](#_Toc159928406)

[Figure S4. Median total log ensovibep serum concentration over scheduled timepoints* (PK analysis set) 19](#_Toc159928407)

[Supplemental Tables 20](#_Toc159928408)

[Table S1. Proportion of patients with anti–SARS-CoV-2 antibodies at baseline and at Day 91 (full analysis set) 20](#_Toc159928409)

[Table S2. Adjusted ANCOVA model of time-weighted change from baseline in log_10_ SARS-CoV-2 viral load through Day 8 in patients at high risk versus those not at high risk of progression to severe COVID-19 and by baseline log_10_ SARS-CoV-2 viral load 21](#_Toc159928410)

[Table S3. Mixed Model for Repeated Measures (MMRM) of SF-36 Mental and Physical Component Summary score by visit (full analysis set) 22](#_Toc159928411)

[Table S4. Complete recovery and patient’s global impression regarding return to pre-COVID-19 health or pre-COVID-19 activities from the long COVID-19 questionnaire (full analysis set) 23](#_Toc159928412)

[Table S5. Summary of pharmacokinetic parameters of total ensovibep serum concentration (pharmacokinetic analysis set) 24](#_Toc159928413)

[Table S6. Common treatment-emergent adverse events by preferred term (≥5 patients; safety set; up to Day 91) 25](#_Toc159928414)

[Table S7. Overview of adverse events of special interest by safety topic (Safety set; up to Day 91) 27](#_Toc159928415)

[Table S8. Anti-drug antibodies positivity at baseline, Day 15, 29, 61, and 91. 29](#_Toc159928416)

# List of investigators

| **Investigators** | **Facility name** |
| --- | --- |
| Istvan Zsolt Varkonyi | Debreceni Egyetem - Klinikai  Kozpont, lnfekto16giai Klinika  Bartok Bela u. 2-26.  H-4031 Debrecen  Hungary |
| A. Venkateshwar Rao | St.Theresa's Hospital  Sanathnagar, Hyderabad  5000 18, Telangana, India |
| Rajesh V. Gosavi | Government Medical College  & Hospital, Medical College  Square Road, Nagpur-  440003,  Maharashtra India |
| Meenakshi Bhattacharya | Government Medical College,  Panchakki road, Aurangabad-  431001, Maharashtra, India |
| Parshottam Koradia | BAPS Pramukh Swami Hospital,  Shri Pramukh Swami Maharaj  Marg, Adajan Char Rasla,  Adajan, Surat - 395009  Gujarat, India |
| Gnana Sundar Y. Raju | Department of General Medicine,  Andhra Medical college, King  George Hospital  Visakhapatnam-530002,  Andhra Pradesh, lndia |
| Poorna Prasad | Shetty's Hospital, Plot Noe 11&12,  12th "F" Main, Kaveri Nagar,  Bommanahalli,  Kodichikkanahalli, Bangalore  - 560068, Karnataka, India |
| Nagalingeswara Kumarasamy | VHS - Infectious Disease Medical Centre,  Rajiv Gandhi Salai,  Taramani, Chennai - 600113,  Tamil Nadu, India |
| Prashant P. Joshi | All India Institute of Medical  Science, MIHAN, Sumthana,  Nagpur-441108, Maharashtra,  India |
| Sheetal K. Kumar | Durgabai Deshmukh Hospital & Research Centre  University road, Vidyanagar,  Hyderabad, 500044,  Telangana, India |
| Marc J. M. Bonten | Department of Neurology  Heidelberglaan 100, Utrecht,  3584 CX, Netherlands |
| Douwe Marcus de Jong | Jongaie Research, Medicross  Pretoria West 1 51 floor, 551  WF Nkomo street, Pretoria,  0183  South Africa |
| Jennifer Rosa | Clinresco Centres (Pty) Ltd,  Central Professional Suites,  20 Central ave, Kempton  Park, 1619, ZA  South Africa |
| Jaco Cornelius Juhl Jurgens | DJW Navorsing, 70 Shannon Rd,  Noordheuwel,  Krugersdorp, 1739, Gauteng,  South Africa |
| Johannes Mattheus Engelbrecht | Suite 21, Block 3, Vergelegen  Mediclinic  Main Road  Somerset West 7130  South Africa |
| Aysha Ebrahim Badat | WCR: Wits Clinical Research,  Chris Hani Baragwanath Hospital,  Chris Hani Road  South Africa |
| Salim Ahmed | Sandton Medical Research  Centre, Wellness on Alon,  49 East Road, Morningside,  Sandton 2196  South Africa |
| Rosie Mngqibisa | Durban International Clinical  Research Site, Enhancing  Care Foundation |
| Martha Bedelu Mekebeb-Reuter | Excellentis Clinical Trial Consultants  72 York Building, 2nd floor,  York Street, George, 6529,  Western Cape, South Africa. |
| Jeffrey Kenneth Kingsley | IACT Health  800 Talbotton Rd  Columbus, GA 31904  United States |
| Joseph A. Boscia | VitaLink Research  501 Roper Mountain Road  Greenville SC 29615  United States |
| Brian Richard Webster | 1124 Gallery Park Blvd  Wilmington, NC 28412  United States |
| George W. Carr | Jefferson City Medical Group,  1241 W Stadium Blvd  Jefferson City  MO 65109  United States |
| Luis Abrishamian | South Bay Clinical Research Institute  520 N Prospect Ave #201  Redondo Beach, CA 90277 |
| Awawu Igbinadolor | Monroe Biomedical Research  34 3 Venus Street  Monroe, North Carolina  28112 United States |
| Manish Jain | Great Lakes Clinical Trials  LLC  5149 N. Ashland Avenue  Chicago, IL 60640  United States |
| Michael Bryan Denenberg | Clinical Research of Rock Hill  370 S. Herlong Avenue, Suite 100  Rock Hill, SC 29732  United States |
| Steven Geller | Centennial Medical Group -  Research Department  8186 Lark Brown Road, Suite 201  Elkridge, MD 21075  United States |
| Bhaktasharan Chimanbhal Patel | Future Innovative Treatments,  LLC  2920 N Cascade Ave, 2nd  Floor, Colorado Springs, CO  80907 United States |
| Alexander Osowa | Gwinnett Research Institute  2805 Hamilton Mill Road  Buford, GA 30519  United States |
| Haresh D. Boghara | Epic Medical Research  106 Plaza Dr ·  Red Oak,  TX 75154 United States |
| Brian J. Bearie | Colton Urgent  Care/Benchmark Research  1181 North Mount Vernon Avenue  Colton, CA 92324 United States |
| Manuel J. Sanchez | Family Practice Center  501 N. Ware Rd.  McAllen, TX 78501 United States |
| Victor Escobar | 1960 Family Practice, PA  837 Cypress Creek Pkwy, Ste 105  Houston, TX 77090 United States |
| Jan H. Westerman | 1280 Summit Drive  Jasper, Alabama 35501  United States |
| Murtaza Mussaji | Fairway Medical Clinic,  4910 Telephone Rd Houston  TX 77087 United States |
| Tewodros Teketel | Zion Urgent Care Clinic  25311 Kingsland Blvd., Ste. 190  Katy, TX 77494 United States |
| Mark E. Kutner | Suncoast Research Group,  LLC  2128 West Flagler Street  Miami, FL 33135 United States |
| Mira Baron | Palm Beach Research Center  2277 Palm Beach Lakes Blvd.  West Palm Beach, FL 33409 United States |
| Robert Godfrey Perry | Panax Clinical Research  14600 NW 60th Avenue, Unit B  Miami Lakes, Florida  United States |
| Andrew Martin | Boward Infectious Disease  and Primary Care, 2825 N  State Road 7  Margate, FL 33063 United States |
| Alejandro Alva | Pacific Neuropsychiatric  Specialists Clinical Research,  LLC 26024 Acero,  Mission Viejo, CA 92691 United States |
| Hector Sotero Rodriguez | Life Spring Research  Foundation, LLC  432 SW 8th Ave Miami FL  33130 United States |
| Lilia Roque Guerrero | Bio-Medical Research, LLC  13226 SW 8th St.  Miami, FL 22184  United States |
| Matthew W. Abinante | Ascada Research  301 W Bastanchury Rd  Fullerton, CA 92835  United States |

# Methods

## Inclusion and exclusion criteria

- Patients eligible for inclusion in this study must have met all the following criteria:
  - Males or females ≥ 18 years of age on the day of inclusion (no upper limit)
  - Presence of two or more of the following COVID-19 symptoms with an onset within 7 days of dosing: feeling hot or feverish, cough, sore throat, low energy or tiredness, headache, muscle or body aches, chills or shivering, and shortness of breath
  - Positive test for SARS-CoV-2 in upper respiratory swab on the day of dosing (rapid antigen test).
  - Understood and agreed to comply with the planned study procedures
  - The patient or legally authorized representative gave signed informed consent
- Patients meeting any of the following criteria were not eligible for inclusion in this the study:
  - Requiring hospitalization at time of screening, or at time of study drug administration
  - Oxygen saturation (SpO_2_) ≤ 93% on room air at sea level or ratio of arterial oxygen partial pressure (PaO_2_ in mmHg) to fractional inspired oxygen (FiO_2_) < 300, respiratory rate ≥ 30 per minute, and heart rate ≥ 125 per minute
  - In India, patients with a respiratory rate ≥ 24 per minute or with an oxygen saturation ≤ 93% on room air (SpO_2_) were not eligible
  - Known allergies to any of the components used in the formulation of the ensovibep or placebo
  - Suspected or proven serious, active bacterial, fungal, viral, or other infection (besides SARS-CoV-2) that in the opinion of the investigator could constitute a risk when taking intervention
  - Any serious concomitant systemic disease, condition, or disorder that, in the opinion of the investigator, should preclude participation in this study
  - Any comorbidity requiring surgery within 7 days of dosing, or that was considered life-threatening within 29 days of dosing
  - Prior or concurrent use of any medication for treatment of COVID-19, including antiviral agents, convalescent serum, or anti-viral antibodies. Purely symptomatic therapies (e.g., over-the-counter cough medications, acetaminophen, and nonsteroidal anti-inflammatory drugs) are permitted. Prior use of steroids for management of COVID-19 symptoms was permitted, provided they could be stopped at study entry based on investigator judgement. Prior vaccination for COVID-19 was permitted
  - Were concurrently enrolled or were enrolled within the last 30 days or within 5 half-lives (whichever is longer) in any other type of medical research judged not to be scientifically or medically compatible with this study
  - Were pregnant or breastfeeding
  - Women of child-bearing potential, defined as all women physiologically capable of becoming pregnant, unless they were using highly effective methods of contraception at the time of dosing and for 11weeks after dosing of study drug. Highly effective contraception methods include:
    - Total abstinence (when this is in line with the preferred and usual lifestyle of the patient). Periodic abstinence (i.e., calendar, ovulation, symptothermal, and postovulation methods) and withdrawal are not acceptable methods of contraception.
    - Female sterilization (have had bilateral surgical oophorectomy [with or without hysterectomy], total hysterectomy, or bilateral tubal ligation at least 6 weeks before taking study treatment). In case of oophorectomy alone, only when the reproductive status of the woman has been confirmed by follow-up hormone level assessment.
    - Male sterilization (at least 6 months prior to screening). The vasectomized male partner should be the sole partner for that patient.
    - Use of oral, injected, or implanted hormonal methods of contraception or placement of an intrauterine device (IUD) or intrauterine system (IUS) or other forms of hormonal contraception that have comparable efficacy (failure rate < 1%), for example hormone vaginal ring or transdermal hormone contraception.
    - In case of use of oral contraception, women should have been stable on the same pill for a minimum of 3 months before taking study treatment.
    - If local regulations deviate from the contraception methods listed above to prevent pregnancy, local regulations apply and will be described in the informed consent form (ICF).
  - Patients in the USA who are at high risk of progression to severe COVID-19 illness or hospitalization (Body mass index ≥ 35; chronic kidney disease; diabetes mellitus; immunosuppressive disease; ongoing immunosuppressive treatment; age ≥ 65 years; or age ≥ 55 years in a patient with cardiovascular disease, or hypertension, or COPD / other chronic respiratory disease) were not to be enrolled in this study as a placebo-controlled study would not have been appropriate in this patient population due to the availability of anti-viral mAbs under EUA in the USA.

## Per-protocol definition of high-risk patients

Patients were considered “high-risk” if they met at least one of the following: body mass index ≥ 35, chronic kidney disease, diabetes mellitus, immunosuppressive disease; (taking) immunosuppressive treatment; were ≥ 65 years of age; or were ≥ 55 years of age with cardiovascular disease, hypertension, chronic obstructive pulmonary disease, or other chronic respiratory disease. Patients not meeting these criteria were regarded as “not high risk.” According to the updated definition of “high risk” issued by the FDA in May 2021, while the study was ongoing, BMI between 25 and 35 was also considered high risk.^1^

## SARS-CoV-2 and antibody testing

Testing SARS-CoV-2 positivity for patient eligibility to the study was done at study sites using rapid antigen tests with nasopharyngeal sample collection: CareStart^TM^ COVID-19 Antigen test (Access Bio Inc) in the US and Panbio^TM^ COVID-19 Ag Rapid Test Device (Abbott) in all the other locations.

All other biomarker tests were performed centrally at Novartis-dedicated laboratories. Central confirmation for SARS-CoV-2 positivity and assessment of viral load for the primary endpoint assessment was performed using quantitative reverse transcriptase PCR (TaqPath™ COVID-19 Combo Kit, ThermoFisher, with calibration curve of Armored RNA Quant™ SARS-CoV-2 Controls, Asuragen). The TaqPath^TM^ COVID-19 Combo Kit targets 3 genes from the orf1a/b, S, and N regions of the virus. The viral copy number was determined using data for the N region only, and reported as viral genome copies/mL.

Whole viral genome sequencing was conducted with Ion AmpliSeq SARS-CoV-2 Research Panel (ThermoFisher) using RNA isolated from same nasopharyngeal samples as for qRT-PCR. Variants were called using the Variant Caller plugin Torrent Variant Caller (TVC) and annotated using the COVID19AnnotateSnpEff plugin. The Phylogenetic Assignment of Named Global Outbreak LINeages (PANGOLIN) software v3.05 with PangoLEARN v2021-06-15 and pango lineages v1.2.13 was used to identify viral lineage.

Anti–SARS-CoV-2 IgG antibodies were tested using a quantitative laboratory developed test at Q2 Solutions based on LIAISON^®^ SARS-CoV-2 S1/S2 IgG assay. For IgM, LIAISON^®^
SARS-CoV-2 IgM assay, with SARS-CoV-2 spike receptor-binding domain (RBD) antigen for coating, was used. Anti–SARS-CoV-2 antibodies were considered as present if test was positive for either IgG or IgM antibodies. Levels of SARS-CoV-2–neutralizing antibodies were tested in a pseudovirus system with recombinant vesicular stomatitis virus (VSV) backbone expressing SARS-CoV-2 spike (S) protein in place of VSV glycoprotein and expressing green fluorescent protein. In the test, patient serum was serially diluted and incubated with recombinant VSV. After neutralization of the serum was allowed to take place (30 min, 37 °C), Vero cells were inoculated with the mixture. Infected cells were fixed with formaldehyde after 7 h and visualized with nuclear stain (DRAQ5). Neutralizing titers were determined based on percent reduction compared to virus control.

**Analysis of the primary end point**

The primary variable for the study is a virologic endpoint defined as time-weighted change from baseline to Day 3, 5, and 8 (2, 4, and 7 days after dosing, respectively) in log10 SARS-CoV-2 viral load in nasopharyngeal swabs.

To calculate the time-weighted change from log10 SARS-CoV-2 viral load through Day 8 for each patient, the equation below is used:

$$\frac{\sum_{i=a}^{b-1} \{0.5\left( Y_{i}+Y_{i+1} \right)\left( t_{i+1}-t_{i} \right)\}}{t_{b}-t_{a}}$$

where Y_i is the change from baseline in log10 SARS-CoV-2 viral load at Visit i, t_i is the time at the Visit i (the actual study day), a is the baseline assessment at Day 1, and b is the last assessment at or prior to Day 8. Viral load values below the lower limit of quantification (LLOQ) will be imputed as 0.5*value of the LLOQ. Viral load values above the upper limit of quantification (ULOQ) will be imputed as value of the ULOQ.

## Multiple Comparison Procedure – Modelling (MCP-Mod) methodology

A generalized MCP-Mod was implemented in a three-step approach: (a) conventional step – covariate-adjusted mean response for each dose; (b) proof-of-concept step – multiple contrast tests; (c) dose-finding step – dose-response model fitting. The target dose was estimated from MCP-Mod for a delta of 0.36 based on the estimated dose-response curve (median of bootstrap estimates). The target dose was the smallest dose among the investigated doses which reached the effect of delta.

## Adverse events of special interest (AESI)

The following represent the list of AESIs for this study:

- A Common Terminology Criteria for Adverse Events grade 2 or higher (i.e., requiring intervention) of:
- Infusion-site reactions
- Hypersensitivity reactions (including anaphylaxis, immune complex mediated hypersensitivity, cytokine release syndrome, and other hypersensitivity reactions)
- Worsening of COVID-19 disease that was reported as a moderate or severe AE per Investigator judgement, with onset within 1 week of study drug administration
- Liver events requiring follow-up
- Renal events requiring follow-up

## Long COVID-19 questionnaire

Patients were asked questions to evaluate the presence and severity of ongoing COVID-19 symptoms, or post–COVID-19 syndrome. This formed part of the clinical assessment alongside the SF-36 questionnaire at Day 29, 61, and 91.

|  | **Questions** | **Response options** |
| --- | --- | --- |
| Respiratory: Breathlessness | Are you having any difficulty **breathing**? | ☐ no, or not more than pre-COVID  ☐ somewhat more than pre-COVID  ☐ a lot more than pre-COVID |
| Respiratory: Cough | Do you have **cough**? | ☐ no, or not more than pre-COVID  ☐ somewhat more than pre-COVID  ☐ a lot more than pre-COVID |
| Systemic: Fatigue | Do you have **low energy**, or feel **tired**? | ☐ no, or not more than pre-COVID  ☐ somewhat more than pre-COVID  ☐ a lot more than pre-COVID |
| Systemic: Post-exertional malaise (physical) | Do your symptoms get worse after **physical activity**?  (This could be immediately after the activity, but also hours later, or the following day) | ☐ I tolerate physical activity like pre-COVID  ☐ I notice a worsening after intense physical activity  ☐ I notice a worsening even after mild physical activity |
| Systemic: Post-exertional malaise (mental) | Do your symptoms get worse after **mental effort, or after stress**?  (This could be immediately after the activity, but also hours later, or the following day) | ☐ I tolerate mental effort or stress like pre-COVID  ☐ I notice a worsening after intense mental effort or stress  ☐ I notice a worsening even after mild mental effort or stress |
| Cognitive: concentration, brain fog | Do you have “brain fog”? (i.e., difficulties in **concentration** or **thinking**)? | ☐ no, or not more than pre-COVID  ☐ somewhat more than pre-COVID  ☐ a lot more than pre-COVID |
| Cognitive: memory | Do you have difficulties to **remember** things? | ☐ no, or not more than pre-COVID  ☐ somewhat more than pre-COVID  ☐ a lot more than pre-COVID |
| Cognitive: communication | Do you have difficulty with finding the right words or understanding others? | ☐ no, or not more than pre-COVID  ☐ somewhat more than pre-COVID  ☐ a lot more than pre-COVID |
| Cardiovascular: palpitations, tachycardia | Do you feel your heartbeat (“palpitations”), and/or do you feel your heart beating too fast? | ☐ no, or not more than pre-COVID  ☐ occasionally, or somewhat more than pre-COVID  ☐ often, or a lot more than pre-COVID |
| Pain: Chest pain or tightness | Do you experience chest pain or chest tightness? | ☐ no, or not more than pre-COVID  ☐ occasionally, or somewhat more than pre-COVID  ☐ often, or a lot more than pre-COVID |
| Pain: Joint pain or muscle aches | Do you experience body pain? (such as joint and/or muscle aches) | ☐ no, or not more than pre-COVID  ☐ occasionally, or somewhat more than pre-COVID  ☐ often, or a lot more than pre-COVID |
| Pain: Headaches | Do you experience headaches? | ☐ no, or not more than pre-COVID  ☐ occasionally, or somewhat more than pre-COVID  ☐ often, or a lot more than pre-COVID |
| Sensory: smell or taste | How is your sense of smell? | ☐ similar to pre-COVID  ☐ somewhat different or worse than pre-COVID  ☐ a lot worse than pre-COVID |
| Sensory: smell or taste | How is your sense of taste? | ☐ similar to pre-COVID  ☐ somewhat different or worse than pre-COVID  ☐ a lot worse than pre-COVID |
| Employment | Have you returned to work? | ☐ I was not employed pre-COVID  ☐ I lost my job  ☐ I am able to work a similar number of hours and at the same level as pre-COVID  ☐ I can only work fewer hours and/or do less demanding tasks compared to pre-COVID  ☐ I currently can’t work at all  ☐ No answer |
| Usual health | Have you returned to your usual health (before your COVID-19 illness)? | ☐ Yes  ☐ No |
| Usual activities | Have you returned to your usual activities (before your COVID-19 illness)? | ☐ Yes  ☐ No |

## Immunogenicity

Blood samples were collected and processed to serum for immunogenicity assessments on Day 1 (pre-dose), 15, 29, 61, and 91. The presence of anti-drug antibodies (ADAs) to ensovibep was assessed using a validated ligand-binding electrochemiluminescence bioanalytical method.^2^

## Pharmacokinetics

Blood samples were collected and processed to serum for pharmacokinetics assessments on Day 1 (pre-dose, 15 min and 90 min), 3, 8, 15, 29, 61, and 91. Pharmacokinetic time points began after intravenous administration, which was defined as the end of the saline flush. Total ensovibep was quantified by a validated liquid chromatography–tandem mass spectrometry (LC-MS/MS) bioanalytical method using a surrogate peptide with a lower limit of quantitation of 0.5 µg/mL. Ensovibep pharmacokinetic parameters were determined by a non-compartmental analysis using Pheonix WinNonlin (Certara).

# Supplemental Figures

## Figure S1. EMPATHY study design


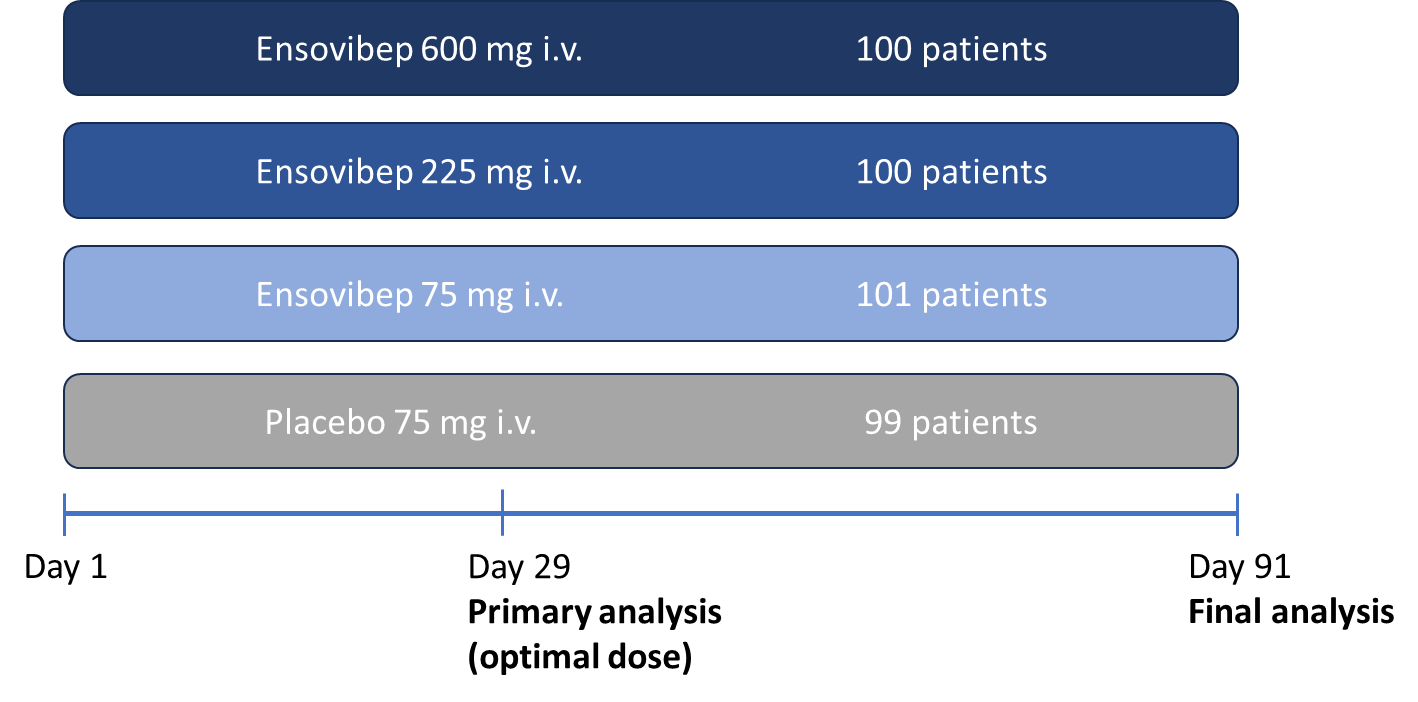


i.v., intravenous.

## Figure S2. Least squares means with 95% confidence intervals and fitted average model (median and 95% percentile interval) for time-weighed change from baseline in log_10_ SARS-CoV-2 viral load through Day 8 (full analysis set)


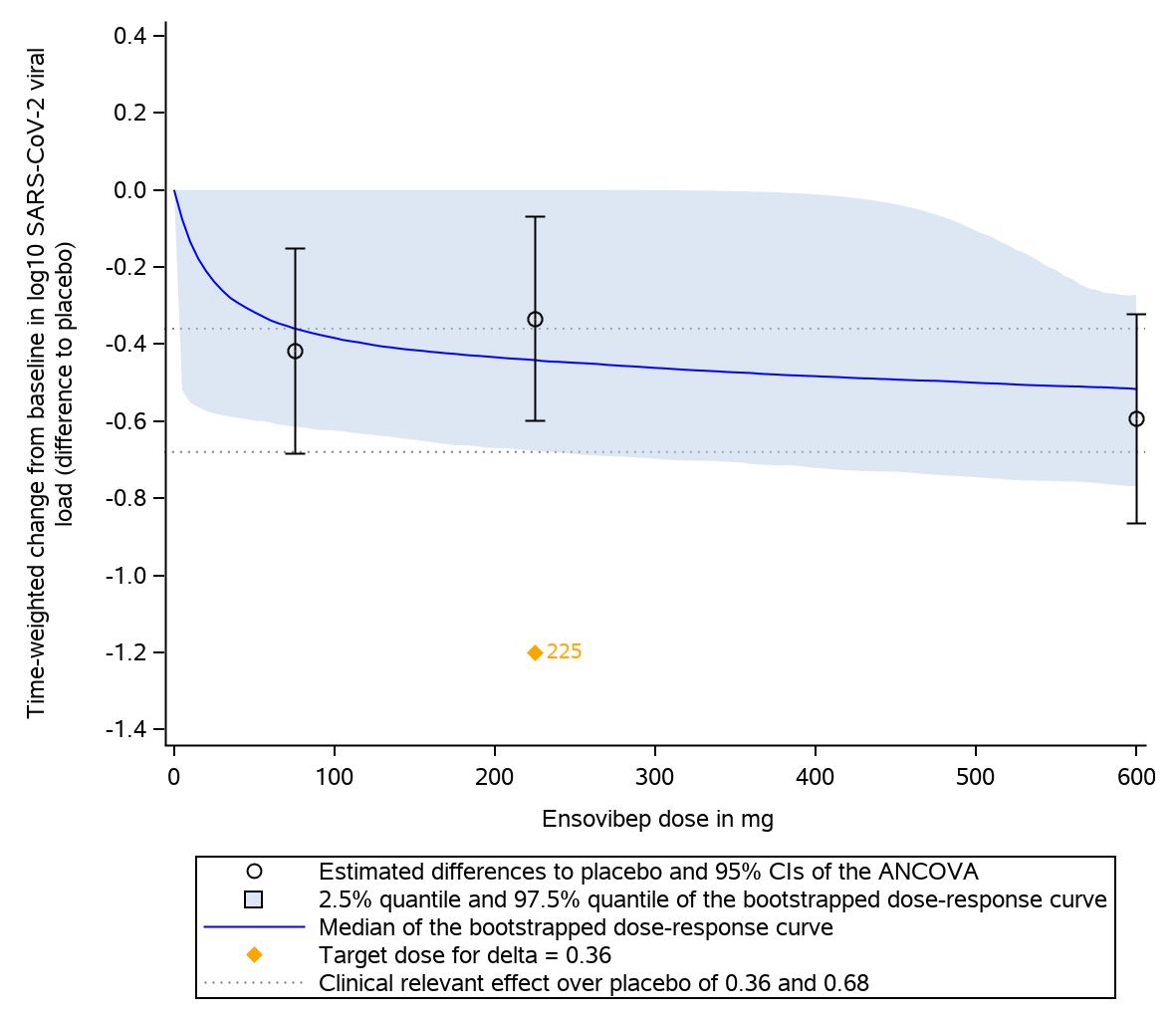

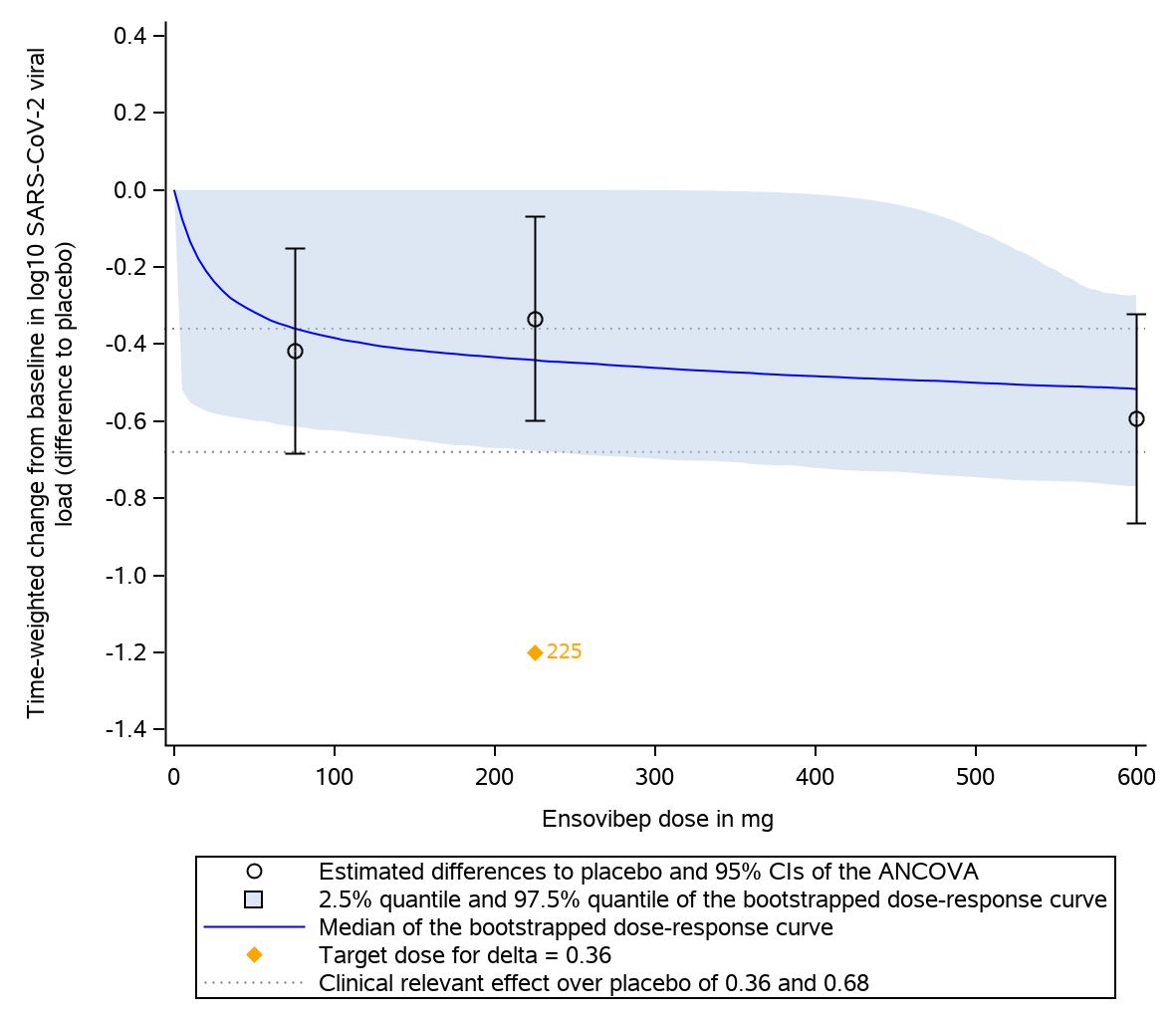


ANCOVA, analysis of covariance; CI, confidence interval.

## Figure S3. Forest plot of estimated treatment differences and associated 95% confidence intervals in time-weighted change from baseline in log_10_ SARS-CoV-2 viral load through Day 8 by subgroups for presence of anti–SARS-CoV-2 antibodies


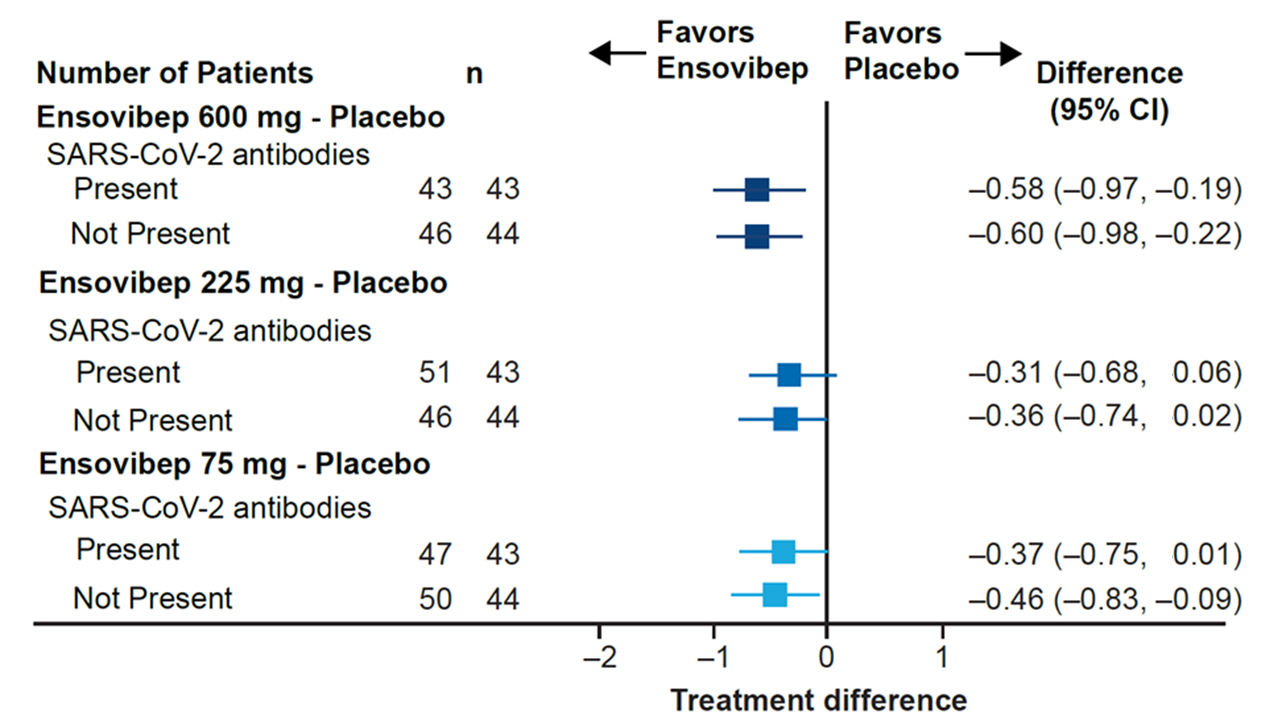


CI, confidence interval.

Data analyzed using ANCOVA, adjusting for Time-weighted change from baseline in log10 SARS-CoV-2 viral load = treatment + baseline log10 SARS-CoV-2 viral load + baseline risk for COVID-19 disease progression + presence of anti-SARS-CoV-2 antibodies at baseline + geographical region. Partial data have been presented at ECCMID, ERS, and IDWeek.^3^

## Figure S4. Median total log ensovibep serum concentration over scheduled timepoints* (PK analysis set)


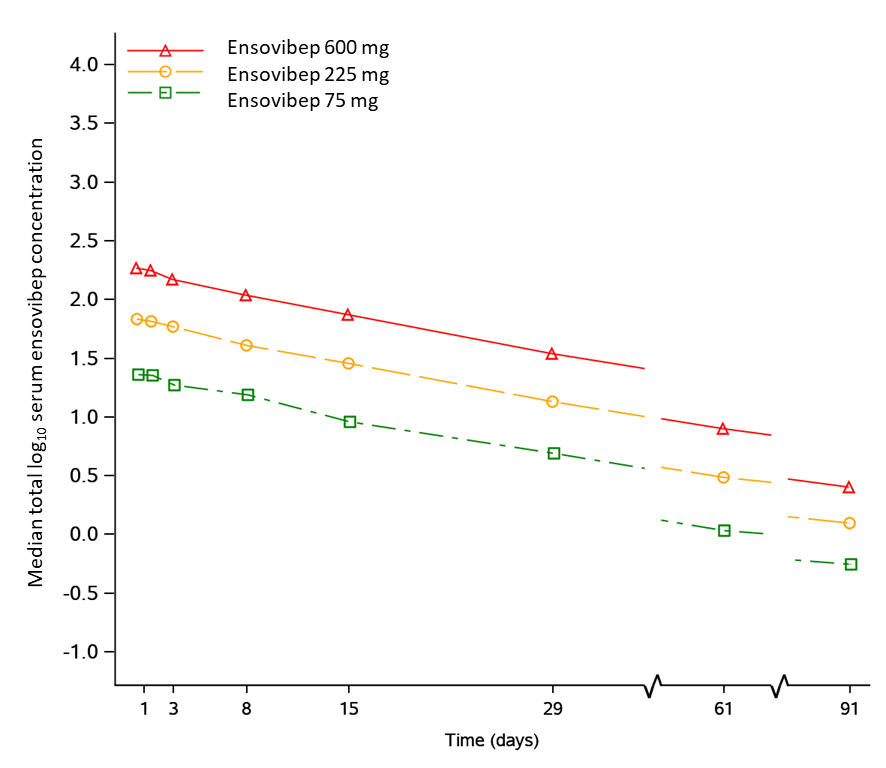


PK, pharmacokinetics.

*At Day 1 scheduled timepoints presented are 15 minutes and 90 minutes post-dose.

# Supplemental Tables

## Table S1. Proportion of patients with anti–SARS-CoV-2 antibodies at baseline and at Day 91 (full analysis set)

| **Antibody test** | **Ensovibep 600 mg** | | **Ensovibep 225 mg** | | **Ensovibep 75 mg** | | **Placebo** |
| --- | --- | --- | --- | --- | --- | --- | --- |
| **Baseline** |  |  | |  | |  | |
| SARS-CoV-2 neutralization assay |  | |  | |  | |  |
| Positive, n/m (%) | 43/97 (44.3) | | 41/97 (42.3) | | 43/99 (43.4) | | 41/99 (41.4) |
| IgG |  | |  | |  | |  |
| Positive, n/m (%) | 43/94 (45.7) | | 49/99 (49.5) | | 44/101 (43.6) | | 38/91 (41.8) |
| IgM |  | |  | |  | |  |
| Positive, n/m (%) | 12/94 (12.8) | | 16/99 (16.2) | | 18/101 (17.8) | | 14/92 (15.2) |
| **Day 91** |  |  | |  | |  | |
| SARS-CoV-2 neutralization assay |  |  | |  | |  | |
| Positive, n/m (%) | 88/94 (93.6) | | 78/89 (87.6) | | 83/95 (87.4) | | 78/91 (85.7) |
| IgG |  | |  | |  | |  |
| Positive, n/m (%) | 78/87 (89.7) | | 78/85 (91.8) | | 77/86 (89.5) | | 76/81 (93.8) |
| IgM |  | |  | |  | |  |
| Positive, n/m (%) | 13/89 (14.6) | | 10/89 (11.2) | | 18/91 (19.8) | | 26/88 (29.5) |

m, number of patients with evaluable data.

## Table S2. Adjusted ANCOVA model of time-weighted change from baseline in log_10_ SARS-CoV-2 viral load through Day 8 in patients at high risk versus those not at high risk of progression to severe COVID-19 and by baseline log_10_ SARS-CoV-2 viral load

| **Subgroup** | **Treatment** | **n** | **LS Mean** | **(SE)** | **(95% CI)** | **p-value** |
| --- | --- | --- | --- | --- | --- | --- |
| **By risk of progression to severe COVID-19** | | | | | | |
| Not at high risk | Ensovibep 600 mg (N=85) | 76 | -0.41 | (0.147) | ( -0.70, -0.12) | 0.005 |
|  | Ensovibep 225 mg (N=84) | 81 | -0.26 | (0.144) | ( -0.54, 0.03) | 0.078 |
|  | Ensovibep 75 mg (N=79) | 77 | -0.27 | (0.147) | ( -0.55, 0.02) | 0.072 |
| At high risk | Ensovibep 600 mg (N=15) | 13 | -1.68 | (0.362) | ( -2.39, -0.97) | <.001 |
|  | Ensovibep 225 mg (N=16) | 16 | -0.84 | (0.344) | ( -1.52, -0.16) | 0.015 |
|  | Ensovibep 75 mg (N=22) | 20 | -1.23 | (0.329) | ( -1.87, -0.58) | <.001 |
| **By baseline log_10_ SARS-CoV-2 viral load** | | | | | | |
| <6 | Ensovibep 600 mg (N=34) | 32 | -0.22 | (0.247) | ( -0.70, -0.27) | 0.380 |
|  | Ensovibep 225 mg (N=36) | 36 | -0.13 | (0.238) | ( -0.60, 0.34) | 0.578 |
|  | Ensovibep 75 mg (N=34) | 34 | -0.34 | (0.242) | ( -0.81, 0.14) | 0.163 |
| ≥6 | Ensovibep 600 mg (N=61) | 57 | -0.77 | (0.203) | ( -1.17, -0.37) | <.001 |
|  | Ensovibep 225 mg (N=62) | 61 | -0.42 | (0.201) | ( -0.81, -0.02) | 0.038 |
|  | Ensovibep 75 mg (N=63) | 63 | -0.53 | (0.200) | ( -0.92, -0.14) | 0.009 |

## Table S3. Mixed Model for Repeated Measures (MMRM) of SF-36 Mental and Physical Component Summary score by visit (full analysis set)

| **Day** | Ensovibep 75 mg | Ensovibep 225 mg | Ensovibep 600 mg |
| --- | --- | --- | --- |
| **SF-36 Physical Component Summary** | | | |
| Day 29  LS mean (95% CI)  p-value | -1.7 (-4.8, 1.3)  0.262 | -0.7 (-3.7, 2.4)  0.665 | -0.6 (-3.7, 2.5)  0.698 |
| Day 61  LS mean (95% CI)  p-value | -1.2 (-4.0, 1.6)  0.387 | -0.3 (-3.1, 2.5)  0.847 | 1. (-2.8, 2.9)   0.979 |
| Day 91  LS mean (95% CI)  p-value | -1.0 (-3.8, 1.7)  0.467 | -1.1 (-3.8, 1.7)  0.443 | -0.7 (-3.4, 2.1)  0.645 |
| **SF-36 Mental Component Summary** | | | |
| Day 29  LS mean (95% CI)  p-value | 0.1 (-3.4, 3.7)  0.935 | -0.4 (-3.9, 3.1)  0.826 | 0.6 (-3.0, 4.1)  0.759 |
| Day 61  LS mean (95% CI)  p-value | -2.4 (-5.8, 0.9)  0.156 | -1.4 (-4.8, 1.9)  0.402 | -2.9 (-6.3, 0.5)  0.099 |
| Day 91  LS mean (95% CI)  p-value | -0.8 (-4.1, 2.5)  0.637 | -1.4 (-4.8, 1.9)  0.395 | -1.6 (-5.0, 1.8)  0.364 |

CI, confidence interval; LS, least square.

## Table S4. Complete recovery and patient’s global impression regarding return to pre-COVID-19 health or pre-COVID-19 activities from the long COVID-19 questionnaire (full analysis set)

|  | **Ensovibep 600 mg**  **n/N (%)** | | **Ensovibep 225 mg**  **n/N (%)** | | **Ensovibep 75 mg**  **n/N (%)** | | **Placebo**  **n/N (%)** |
| --- | --- | --- | --- | --- | --- | --- | --- |
| **Complete recovery* by visit** |  |  | |  | |  | |
| Day 29 | 24/87 (27.6) | | 26/85 (30.6) | | 22/90 (24.4) | | 21/83 (25.3) |
| Day 61 | 30/82 (36.6) | | 32/84 (38.1) | | 26/84 (31.0) | | 35/90 (38.9) |
| Day 91 | 36/79 (45.6) | | 36/82 (43.9) | | 25/82 (30.5) | | 34/84 (40.5) |
| **Return to usual health (as before COVID-19 illness)^†^** | | | | | | | |
| Day 29 | 68/87 (78.2) | | 64/85 (75.3) | | 62/90 (68.9) | | 63/83 (75.9) |
| Day 61 | 70/82 (85.4) | | 69/84 (82.1) | | 69/84 (82.1) | | 76/90 (84.4) |
| Day 91 | 68/79 (86.1) | | 68/82 (82.9) | | 69/82 (84.1) | | 71/84 (84.5) |
| **Return to usual activities (as before COVID-19 illness)^†^** | | | | | | | |
| Day 29 | 76/87 (87.4) | | 73/85 (85.9) | | 74/90 (82.2) | | 72/83 (86.7) |
| Day 61 | 76/82 (92.7) | | 73/84 (86.9) | | 74/84 (88.1) | | 83/90 (92.2) |
| Day 91 | 72/79 (91.1) | | 73/82 (89.0) | | 74/82 (90.2) | | 75/84 (89.3) |

*All questions per visit were answered with 'like pre-COVID'.

^†^Individual question answered by the patient.

n: number of patients with 'like pre-COVID' as an answer for all questions at the respective visit (complete recovery); number of patients with ‘Yes’ as an answer (return to pre-COVID-19 health or pre-COVID-19 activities).

N: number of patients with an answer to the questionnaire at the respective visit (complete recovery and return to pre-COVID-19 health or pre-COVID-19 activities).

## Table S5. Summary of pharmacokinetic parameters of total ensovibep serum concentration (pharmacokinetic analysis set)

| **PK parameter** |  | **Ensovibep 600 mg N=95** | **Ensovibep 225 mg N=94** | **Ensovibep 75 mg N=97** |
| --- | --- | --- | --- | --- |
| **C_max_, μg/mL** |  |  |  |  |
| n |  | 94 | 90 | 93 |
| Mean (SD) |  | 193 (50.9) | 72.9 (19.5) | 27.1 (13.6) |
| **AUC infinity, h*μg/mL** |  |  |  |  |
| n |  | 87 | 77 | 82 |
| Mean (SD) |  | 79400 (25500) | 29200 (9500) | 10600 (3850) |
| **AUC%extrap, %**  n  Mean (SD) |  | 90  5.45 (12.8) | 81  7.36 (12.7) | 80  12.0 (14.7) |
| **T_1/2_ Lambda z, h** |  |  |  |  |
| n |  | 89 | 81 | 83 |
| Mean (SD) |  | 303 (115) | 323 (141) | 330 (114) |

AUC, area under the curve; C_max_, maximum concentration; SD, standard deviation, T_1/2_, half life; AUC%extrap, area under the curve extrapolated as a percentage of the total

## Table S6. Common treatment-emergent adverse events by preferred term (≥5 patients; safety set; up to Day 91)

| **Preferred term** | **Ensovibep 600 mg N=100 n (%)** | **Ensovibep 225 mg N=98 n (%)** | **Ensovibep 75 mg N=102 n (%)** | **Ensovibep total N=300 n (%)** | **Placebo N=100 n (%)** |
| --- | --- | --- | --- | --- | --- |
| At least one treatment-emergent adverse event | 49 (49.0) | 42 (42.9) | 37 (36.3) | 133 (44.3) | 54 (54.0) |
| Alanine aminotransferase increased | 7 (7.0) | 3 (3.1) | 1 (1.0) | 11 (3.7) | 2 (2.0) |
| COVID-19 | 6 (6.0) | 3 (3.1) | 1 (1.0) | 10 (3.3) | 4 (4.0) |
| Aspartate aminotransferase increased | 6 (6.0) | 3 (3.1) | 1 (1.0) | 10 (3.3) | 2 (2.0) |
| Blood creatinine increased | 6 (6.0) | 4 (4.1) | 3 (2.9) | 13 (4.3) | 6 (6.0) |
| Fibrin D dimer increased | 5 (5.0) | 2 (2.0) | 4 (3.9) | 11 (3.7) | 3 (3.0) |
| Lipase increased | 6 (6.0) | 0 | 3 (2.9) | 9 (3.0) | 1 (1.0) |
| Headache | 2 (2.0) | 2 (2.0) | 4 (3.9) | 8 (2.7) | 1 (1.0) |
| Nasopharyngitis | 5 (5.0) | 1 (1.0) | 3 (2.9) | 9 (3.0) | 5 (5.0) |
| Neutropenia | 3 (3.0) | 1 (1.0) | 4 (3.9) | 8 (2.7) | 1 (1.0) |
| Amylase increased | 4 (4.0) | 1 (1.0) | 1 (1.0) | 6 (2.0) | 2 (2.0) |
| Gamma-glutamyltransferase increased | 3 (3.0) | 1 (1.0) | 3 (2.9) | 7 (2.3) | 1 (1.0) |
| Vomiting | 3 (3.0) | 2 (2.0) | 1 (1.0) | 6 (2.0) | 1 (1.0) |
| Activated partial thromboplastin time prolonged | 3 (3.0) | 1 (1.0) | 1 (1.0) | 5 (1.7) | 1 (1.0) |
| Blood creatine phosphokinase increased | 3 (3.0) | 1 (1.0) | 1 (1.0) | 5 (1.7) | 1 (1.0) |
| COVID-19 pneumonia | 2 (2.0) | 3 (3.1) | 0 | 5 (1.7) | 5 (5.0) |
| Hypertension | 1 (1.0) | 2 (2.0) | 2 (2.0) | 5 (1.7) | 0 |
| Nausea | 3 (3.0) | 1 (1.0) | 0 | 4 (1.3) | 2 (2.0) |

## Table S7. Overview of adverse events of special interest by safety topic (Safety set; up to Day 91)

| **Safety topic**  **Preferred term** | Ensovibep 600 mg N=100 n (%) | Ensovibep 225 mg N=98 n (%) | Ensovibep 75 mg N=102 n (%) | Ensovibep total N=300 n (%) | Placebo N=100 n (%) |
| --- | --- | --- | --- | --- | --- |
| At least one treatment-emergent AE of special interest | 20 (20.0) | 9 (9.2) | 7 (6.7) | 36 (12.0) | 18 (18.0) |
| Worsening of COVID-19 following administration | 5 (5.0) | 4 (4.1) | 1 (1.0) | 10 (3.3) | 7 (7.0) |
| COVID-19 | 3 (3.0) | 1 (1.0) | 1 (1.0) | 5 (1.7) | 2 (2.0) |
| COVID-19 pneumonia | 2 (2.0) | 3 (3.1) | 0 | 5 (1.7) | 5 (5.0) |
| Hepatic events including investigations | 8 (8.0) | 1 (1.0) | 1 (1.0) | 10 (3.3) | 5 (5.0) |
| Alanine aminotransferase increased | 4 (4.0) | 1 (1.0) | 0 | 5 (1.7) | 2 (2.0) |
| Aspartate aminotransferase increased | 2 (2.0) | 1 (1.0) | 0 | 3 (1.0) | 1 (1.0) |
| Blood bilirubin increased | 1 (1.0) | 0 | 1 (1.0) | 2 (0.7) | 0 |
| Hepatic enzyme increased | 1 (1.0) | 0 | 0 | 1 (0.3) | 2 (2.0) |
| Renal events including investigations | 5 (5.0) | 4 (4.1) | 3 (2.9) | 12 (4.0) | 6 (6.0) |
| Blood creatinine increased | 5 (5.0) | 4 (4.1) | 3 (2.9) | 12 (4.0) | 6 (6.0) |
| Hypersensitivity (other than administration site reactions) | 2 (2.0) | 0 | 1 (1.0) | 3 (1.0) | 1 (1.0) |
| Infusion-related reaction | 0 | 0 | 1 (1.0) | 1 (0.3) | 0 |
| Rash | 1 (1.0) | 0 | 0 | 1 (0.3) | 0 |
| Rash maculo-papular | 1 (1.0) | 0 | 0 | 1 (0.3) | 1 (1.0) |
| Acute respiratory failure | 0 | 0 | 0 | 0 | 1 (1.0) |
| Hypersensitivity vasculitis (including unspecified rash) | 2 (2.0) | 0 | 1 (1.0) | 3 (1.0) | 1 (1.0) |
| Petechiae | 0 | 0 | 1 (1.0) | 1 (0.3) | 0 |
| Rash | 1 (1.0) | 0 | 0 | 1 (0.3) | 0 |
| Rash maculo-papular | 1 (1.0) | 0 | 0 | 1 (0.3) | 1 (1.0) |
| A patient with multiple AEs with the same topic was counted only once for that topic. A patient with multiple AEs with the same preferred term was counted only once for that preferred term. A preferred term can appear in more than one topic. All adverse events starting on or after the start of study drug infusion were considered treatment emergent. | | | | | |

AE, adverse event.

## Table S8. Anti-drug antibodies positivity at baseline, Day 15, 29, 61, and 91.

| **Day** | Ensovibep 75 mg | Ensovibep 225 mg | Ensovibep 600 mg |
| --- | --- | --- | --- |
| Baseline, n/N (%) | 4/100 (4.0) | 10/97 (10.3) | 6/95 (6.3) |
| Day 15, n/N (%) | 41/92 (44.6) | 44/88 (50.0) | 45/96 (46.9) |
| Day 29, n/N (%) | 54/96 (56.3) | 60/89 (67.4) | 65/92 (70.7) |
| Day 61, n/N (%) | 52/90 (57.8) | 68/90 (75.6) | 68/94 (72.3) |
| Day 91, n/N (%) | 52/95 (54.7) | 59/89 (66.3) | 68/94 (72.3) |

**References:**

1. The U.S. Food and Drug Administration. Coronavirus (COVID-19) Update: May 21, 2021. (<https://www.fda.gov/news-events/press-announcements/coronavirus-covid-19-update-may-21-2021>).
2. Ter Avest M, Langemeijer SMC, Blijlevens NMA, et al. Dose optimalization of subcutaneous ravulizumab is predicted to yield significant savings and to improve patient friendliness. Br J Clin Pharmacol. 2023;89(3):1211–1215.
3. Abrishamian L, Bonten M, Chandra R, et al. Ensovibep antiviral activity in ambulatory patients with COVID-19 is independent of baseline anti-SARS-CoV-2 antibodies and exhibits minimal selective pressure – Results from the placebo-controlled EMPATHY trial. Poster presented at IDWeek 2022 Washington DC, USA 2022.
